# Persistence and Availability of Web Services in Computational Biology

**DOI:** 10.1371/journal.pone.0024914

**Published:** 2011-09-22

**Authors:** Sebastian J. Schultheiss, Marc-Christian Münch, Gergana D. Andreeva, Gunnar Rätsch

**Affiliations:** 1 Machine Learning in Biology Research Group, Friedrich Miescher Laboratory of the Max Planck Society, Tübingen, Germany; 2 Wilhelm Schickard Institute for Computer Science, University of Tübingen, Tübingen, Germany; Wayne State University, United States of America

## Abstract

We have conducted a study on the long-term availability of bioinformatics Web services: an observation of 927 Web services published in the annual *Nucleic Acids Research Web Server Issues* between 2003 and 2009.

We found that 72% of Web sites are still available at the published addresses, only 9% of services are completely unavailable. Older addresses often redirect to new pages. We checked the functionality of all available services: for 33%, we could not test functionality because there was no example data or a related problem; 13% were truly no longer working as expected; we could positively confirm functionality only for 45% of all services.

Additionally, we conducted a survey among 872 *Web Server Issue* corresponding authors; 274 replied. 78% of all respondents indicate their services have been developed solely by students and researchers without a permanent position. Consequently, these services are in danger of falling into disrepair after the original developers move to another institution, and indeed, for 24% of services, there is no plan for maintenance, according to the respondents.

We introduce a Web service quality scoring system that correlates with the number of citations: services with a high score are cited 1.8 times more often than low-scoring services. We have identified key characteristics that are predictive of a service's survival, providing reviewers, editors, and Web service developers with the means to assess or improve Web services. A Web service conforming to these criteria receives more citations and provides more reliable service for its users.

The most effective way of ensuring continued access to a service is a persistent Web address, offered either by the publishing journal, or created on the authors' own initiative, for example at http://bioweb.me. The community would benefit the most from a policy requiring any source code needed to reproduce results to be deposited in a public repository.

## Introduction

In 2003, the journal *Nucleic Acids Research* (NAR) published its first *Web Server Issue* in an open-access format. This special issue on Web services that perform “useful computations” was described in its editorial as the “natural companion” to the annual, then already decade-old Database Issue [1]. The peer-reviewed contributions consisted of 131 of the most widely known, freely accessible Web services from the years before 2003, which is why the services in this issue are of an exceptional quality. There are many benefits to studying this rather compact but very well-defined collection of services. Authors are expressly allowed to re-publish their service in the *Web Server Issue* after a hiatus of two years, if they can give the number of citations or other measures of community impact for their service, to support its re-publication [2]. In these special issues, we find the most widely known Web services that computational biology has to offer.

In our study, performed during 2009 and 2010, we determined how many of the published Web services from the *Web Server Issues* were still available. We define the term Web service as an application that is available on a specific server over the Internet using a fixed Web address, accessed via a Web browser. Many scientists have relied on these services for data analysis and many articles have been published using results from one of these services. If a service becomes unavailable, results that are based on its output become irreproducible.

In the minds of most computational biologists, Web services are unreliable at best. There is a perception that most services become unavailable quickly after publication or cease to function; at the same time, authors are reluctant to share their source code, or even to help out with technical issues of the service. This is usually anecdotal evidence; successful vs. failed attempts to use someone else's software are rarely offset against each other [3].

We set out to take stock of this curated data set of NAR special issues and to find out how much truth really lies in these stereotypes. Clearly, an analysis of the whole universe of published bioinformatics software would be a monumental task, so we kept to the NAR *Web Server Issues*, a more manageable data set of 927 services (Table 1). Any results we obtain set an upper bound for a larger cross-section of bioinformatics software and in many cases show an idealized picture. Submissions to journals with fewer requirements and restrictions are bound to be of an overall lower availability than the ones presented here.

**Table 1:** Key statistics for this study, across all NAR Web Server Issues from 2003 to 2009.

Description	Number
Total number of services analyzed	927
Total number of publications	913
Total number of citations in PubMed Central	12157
Total number of countries hosting services	39
Total number of institutions hosting services	322
Number of authors contacted	872
Number of responses to author's survey	274

There is a difference between the number of publications vs.services because some publications describe two different services, without becoming a collection.

The Bioinformatics Links Directory lists 1,247 links to bioinformatics “tools and resources,” excluding 448 databases [4], [5]. We thus coverered 74% of the Links Directory with the 927 services in this study (Table 1).

The goal of our study is to identify properties of a service that are indicative of long-lasting availability. While it is true that most services will eventually be superseded by newer ones, they should be available long enough to allow a comparative, independent evaluation: Does the new service really outperform the older one for all inputs?

A service should not have to disappear while it is still useful. Eventually, some data formats created by specific methods will no longer be widely used, and along with them, the standard analysis method will become obsolete. However, “stunning results can be obtained using decade-old data” [6].

Even for services that are still actively used, their maintainers struggle with required but deprecated software libraries, unreachable original developers who have long since moved on to other projects and institutions, and funding that is running out.

Several initiatives are underway to alleviate these problems, but most of them are targeted towards data or “biological information,” not analysis methods, for instance the projects ELIXIR [7] or BioSapiens [8] by the European Union. The European Bioinformatics Institute (EBI) in the United Kingdom hosts a large number of tools and services for computational biologists [9], the National Center for Biotechnology Information (NCBI) in the United States and other institutions also provide an array of tools on their Web sites [10], [11]. To our knowledge, no institution has a formalized way for adopting software written by someone else, which is unfortunate. In response to our inquiry, the NCBI help desk told us that “software maintenance is very labor intensive and providing the service to the public will require extra hardware resources. Both of which NCBI does not have” [12].

We provide our results in an open-access format. Because many researchers are still actively working on their service, our timing may have been unfortunate and we always encountered one service when it was offline. However, this is also the reality for many researchers wanting to access a certain server. They are most likely not going to try more than three times over the course of a year. The data to this article is intended to be shared with everyone and corrections to the record are very welcome.

## Results and Discussion

### Availability Study

#### Examples of Successful Web Services

**Figure 1:**
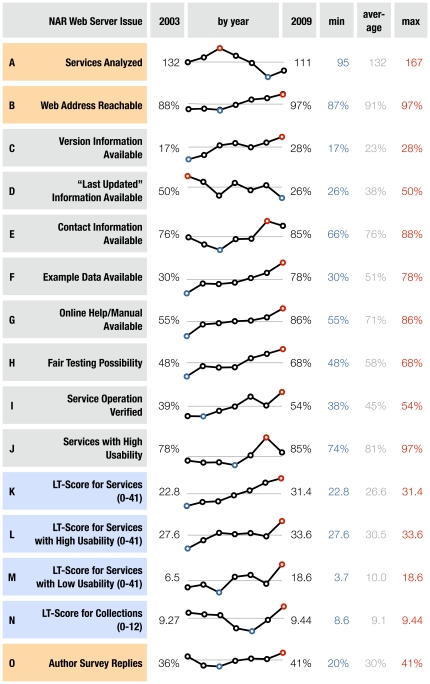
Charts for many criteria used to evaluate the services, by year of publication. This figure shows numerical values and sparklines [27] for the criteria of every year the NAR *Web Server Issue* was published, listing explicitly the values for 2003, 2009, the minimum value of these years in blue (labeled min), the arithmetic mean in gray (labeled mean) as a straight line, and the maximum number in red (labeled max). (A) *Services analyzed* lists the absolute number of services we extracted from that issue of NAR. Some publications describe a whole collection of services, which were not evaluated individually, but rather by criteria apt for collections, and appear as a single service in the graph. (B) *Web address reachable* is a relative number of URLs that did not return an error message when accessed in a browser (as described in [3]), but may contain services that are not operational while still displaying their regular Web page. (C)–(G) We tried to locate *version information*, *“last updated” information*, *contact information*, *example data*, and *online help/manual*, on the service's Web page to the best of our abilities. As the requirements for submissions to NAR *Web Server Issues* tightened, we see an increase in these numbers, except for the version information, which becomes pertinent as the Web service ages. (H) For services to give us a *fair testing possibility*, we required either easily obtainable example data or standard file types such as FASTA, PDB, GFF, etc.(cf. Methods). (I)–(J) The percentage reported in *service operation verified* is taken from the total number of services in that issue. We assigned usability scores from zero to three, *services with high usability* score either two or three. A high score is assigned to services with clearly arranged user interface widgets, the presence of default values and easily accessible help and usage information. It is low for services with strong restrictions on input data and crowded, unclear user interfaces without documentation. (K) The *LT-Score* is calculated for every service, on a range from zero to 41, and zero to twelve for every collection of services (see Methods). (L)–(M) As the *LT-Score for services with high usability* and *low usability* show, services with user interfaces that are well-arranged also have an above average LT-Score, and services with unclear interfaces score significantly lower. (N) The *LT-Score for collections* is quite constant over all issues. (O) The number of authors that participated in the survey is reflected in *Author Survey Replies*. Not surprisingly, authors from more recent services are more likely to respond, not least because their email addresses listed in the publication are still current. The higher number of replies for 2003 reflects the care and commitment the services from this issue have received.

**Figure 2:**
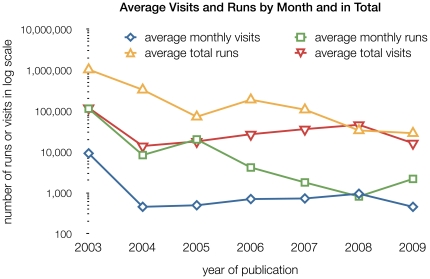
Average number of visits to the Web services and average runs, both by month and in total, in log scale. There is a clear exception for services in the 2003 issue, visible in the number of runs per month: These services are still heavily used and have amassed a very high visitor and run total. 157 of 274 respondents (57%) answered the question about monthly data and 137 (50%) also stated total estimates. The blue, diamond-shaped symbols illustrate how newer services usually have a higher number of monthly visits, declining over time as the services become used less frequently. With the exception of the 2008 average, visitors also seem to use services more than once per visit. This is reflected both in the monthly and in the total numbers.

The Web services introduced in the NAR *Web Server Issue* 2003 are good examples for long-term availability. Here, the readers of this special issue are (re-)introduced to 131 services (cf. Figure 1 A) that have been used for years and that continue to draw large numbers of users. The average monthly visitor count and estimated total count of the 2003 services are orders of magnitude above the ones from later years, as shown in Figure 2. In that sense, the 2003 issue is an exception, because it contains some of the most well-known and highly cited bioinformatics Web services that have been published before 2003 and are still heavily in use today.

#### The Long-term Score: Predictors of Persistence

In the issues following 2003, newer services have been published. Some are already unavailable today (see Figure 1 B). Over the course of the years, the editors tightened the submission requirements, demanding a functional Web service that provides example data, help/tutorial pages, description of input and output formats, and proscribing any kind of registration, login or sign-up [2]. As time progresses, these criteria are fulfilled by more and more submitted Web services (see Figure 1 C–G). We checked for all of these criteria in our study, and added version, update, and contact information to our checklist in Table 2. Using all these criteria, we created the Long-Term-Score (LT-Score), allowing anyone to assess a Web service's compliance with these best-practice criteria. The scoring function for the calculation of the LT-Score is a sum. All scores from this table sum up to a maximum of 41 points for services; for collections of several services only some of the qualities are evaluated, putting their maximum at twelve points.

**Table 2:** Scoring function and qualities analyzed for the LT-Score.

#	Qualities analyzed	yes	no	special
1	Web address available	2	0	–
2	Version information available	1	0	–
3	Hosting country could be determined	1	0	–
4	Hosting institution could be determined	1	0	–
5	Last updated information available	1	0	–
6	Contact information available	3	0	–
7	High usability^a^	2, 3	0, 1	–
8	Registration not required^b^	3	0	1
9	Download not required	3	0	–
10	Example data available	4	0	–
11	Fair testing possibility^c^	5	0	2
12	Service is functional^d^	10	0	4
	LT-Score for services (all characteristics)	41	0	–
	LT-Score for collections (characteristics 1–7)	12	0	–

The scoring function for the calculation of the LT-Score is a sum: to score a service, the qualities listed in the table are evaluated; all scores sum up to a maximum of 41 points for services; for collections of several services only the qualities one through seven are evaluated and summed up to a maximum of twelve points.

a Regular usability results in two points, exceptional usability is awarded with three points. One point is given to services with low usability, and zero if the service was unavailable.

b If the registration is limited to an email address for reporting results, one point is awarded.

c For a fair testing possibility, we require either easily obtainable example data or only standard file types such as FASTA, PDB, GFF, etc. for the input. See Methods for more information. Two points were awarded if we were unable to determine whether we had a fair testing possibility or not.

d If we were unable to determine functionality due to a lacking fair testing possibility, four points were awarded, while clearly non-functional services received zero points.

Over the years, the LT-Score has increased constantly (see Figure 1 K), for which credit goes to the reviewers and editors of NAR for enforcing the submission instructions. More details about this scoring system and the rating of usability can be found in the Methods section and in Table 2.

**Figure 3:**
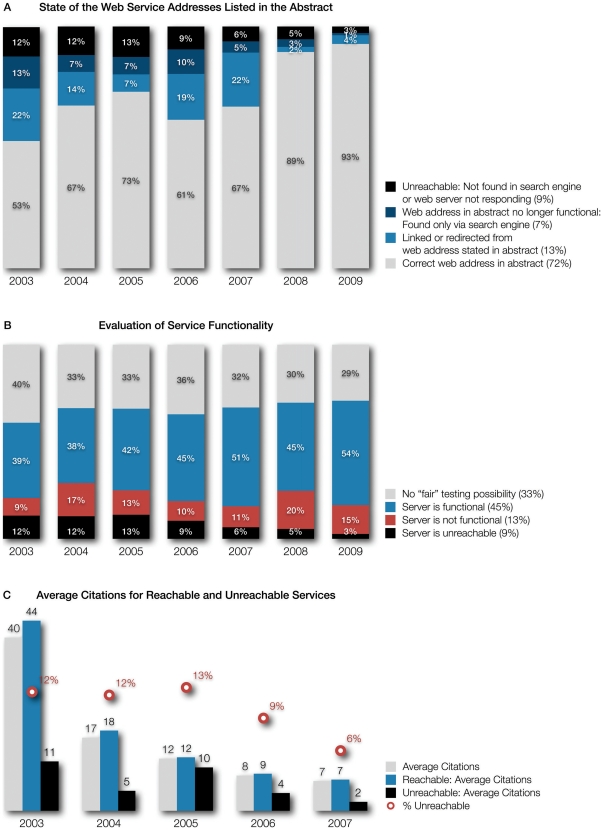
Changes in services' Web address, functionality, and citations plotted by publication year. (**A**) The state of all Web service addresses listed in the abstracts. We extracted the services' Web addresses from the NAR *Web Server Issue* abstracts and entered them into a Web browser to check for inconsistencies. We noted that, for many pages, the original published address is no longer current. The browser is either redirected transparently or a static link on the page informs the visitor of the address change (light blue). While this is a well-meant gesture, eventually, the Web server performing the redirect will be replaced or shut off and the link will appear dead. We therefore also searched for all Web services with dead links using internet search engines to determine if they had moved to a new location (dark blue). 13% of services from 2003 can be found in this way. The percentages of services that are completely unavailable are shown in the black part of the column. Total percentages for each of these measures are given in parenthesis after their description in the key. (**B**) Evaluation of service functionality. We show how many services are not functional even though their Web page is still available (red). This indicates that the software behind the Web pages, the actual Web service, is failing. For users, the reason for that is impossible to determine. A large percentage of services could not be evaluated under the premises of our “fair” testing possibility (cf. Methods): They do not provide example data and on top of that either require very specific file types or complex parameter settings that are not set by default (gray). Functional services make up the largest group, but not the majority (blue). Total percentages for each of these measures are given in parenthesis after their description in the key. (**C**) Comparing the average number of citations for available and unavailable services. Intuitively, unusable services should have a lower number of citations (black). The number of citations is not comparable among years, because older publications have had more time to be cited. Data for the years 2008 and 2009 are not shown, as these publications have not had enough time to be cited (the same trend can be observed, but it is not yet significant). The red numbers show the percentage of services from that year's issue that are unavailable as of October 2010.

Beyond being able to access the Web pages, we checked if the service itself was still functional (shown in Figure 1 I). The NAR submission instructions request a one-click mechanism to try out example data [2]. Whenever we found this on a service's site, we considered it a “fair” testing possibility for service functionality. If there was no such mechanism but a service explained its required input data well and provided downloadable examples, we still considered this a “fair” testing possibility. Finally, we also were in accord with services that required only standard file types (cf. Methods for a list of file types and more details and Figure 1 H for changes over time). We found that 33% of services did not provide us with a “fair” testing possibility (see Figure 3 B). We could verify that the service is operating normally on its example data for 45% of all services published (see Figures 1 I and 3 B). If services no longer work as published, this is most likely because the service's software behind the Web pages is failing. An observant maintainer could immediately tell that there is a problem from the drastic drop in computing resoruce usage or Web page visits.

These numbers of course depend on the time of observation, but judging from the data taken at four different time points over the course of more than one year (June 2009 to October 2010), the number of unavailable services is stable at around 9% (see Figure 3 A). This is much lower than common stereotypes would lead us to believe. Nevertheless, available but nonfunctional services have to be considered as well. Since they are quite hard to detect, we used our definition of a “fair” testing possibility to assess this (see also Table 2), leaving out 33% of services that we were unable to test (see key of Figure 3 B). A total of 13% of services does not deliver the expected results when actually used (see key of Figure 3 B). We thus estimate that at least one quarter of services in computational biology is no longer maintained roughly three years after the latest publication. It is only a question of time until the current Web server where the service runs is replaced or a server software update breaks the functionality of the legacy service.

#### Web Service Addresses

From Figure 3 A, we can learn that, over time, Web service addresses will change. In the whole sample of services analyzed here, none used a persistent URL or a DOI address to refer to their service. Therefore, only 53% of services from the 2003 issue are still available at the same address as the one published in the original manuscript (Figure 3 A). Over time, this will only get worse as old Web servers will be shut down and the services have to be copied to new machines or migrated to another institution. More recent servers are still available at the published address (93% for 2009, Figure 3 A) and only a small number of services has to be found via a search engine or is redirected. There are services that are completely unavailable, most of the time the Web server does not respond to inquiries, and it is difficult to tell when and if it will become available again. As scientific Web servers are usually non-redundant, a certain number of days of downtime is expected, and a study such as this can only be a snapshot in time. When testing 927 services, some of them are expected to be offline on any given day. The numbers for the two latest issues can be explained in this way, but for the previous years, we have to assume that many of the unavailable services will never come back online.

For many authors that need to redirect the published link to a new page, it might be beneficial to invest the time to file a correction with the publishing journal. Then, the updated address would be reflected in the abstract.

#### Collections of Services

As mentioned before, some NAR *Web Server Issue* articles also present collections of services, for instance the article about all tools offered by the European Bioinformatics Institute [9]. In total, 98 or 11% of all manuscripts describe collections of at least three services (see Table 1), for which we did not check whether each individual service was in working order; thus, we have no data on functionality. Most collections are of a very high quality, as they are run by a team of administrators dedicated to this task.

Unfortunately, for developers of new methods that are to be offered as a Web service, setting up their software under the roof of a collection of services is next to impossible [13]. Either the collection is run by a lab or institution that uses the high visibility of their page to promote new developments of their own, or it is funded by a government research agency that does not have the resources to include external services [12].

#### Countries and Institutions as Hosting Providers

When visiting the Web services, we recorded the country and institution hosting the Web site. This is the physical location of the Web server that provides the service to the internet. We used the IP address of the server to determine the country, and the domain name of the Web address to infer the institution.

**Figure 4:**
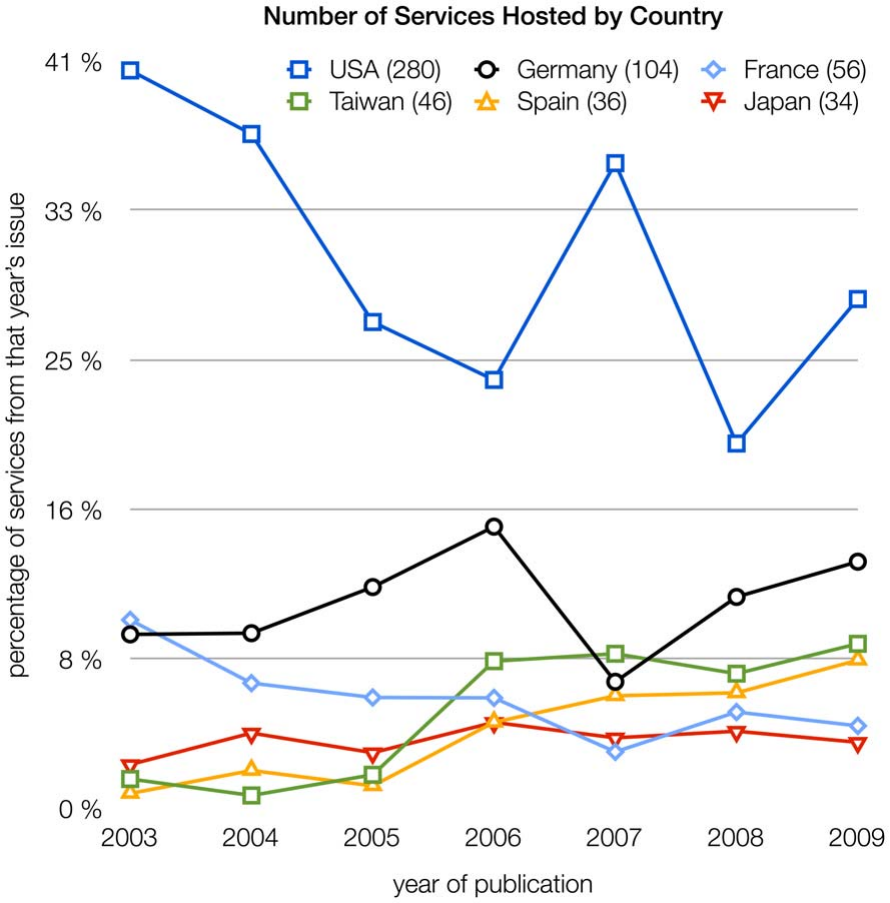
Number of services hosted by country, in percent of services published in that issue. This chart shows six countries with the highest number of hosted services, total numbers are indicated in parenthesis after each country's name. The ten countries on ranks seven to 16 are Canada (33), India (32), United Kingdom (31), China (27), Italy (27), Israel (26), The Netherlands (23), South Korea (20), Switzerland (20), and Singapore (17).

The number of services by country is shown in Figure 4. There is a surprisingly high variance for the number of services hosted in each country over the years: 30% of services are hosted in the United States, but within the seven issues analyzed, we count a maximum of 41% and a minimum of 19% for the United States, a factor of two. The variance increases further for the countries with the second and third most services, Germany and France (Figure 4). Calculating the LT-Score by country leads to even greater variance by year (data not shown). Hosting country and number of services hosted there is thus no indicator for service quality.

In most countries, the institutions hosting the services are quite diverse, but especially in smaller countries, a single institution may be hosting half of that country's services published in the NAR *Web Server Issues*. The top ten ranks of single institutions are shown in Table 3.

**Table 3:** Number of services hosted by a single institution.

Rank	Institution	Country	Services
1	National Chiao Tung University	Taiwan	18
2	Columbia University	USA	17
3	Centro de Investigación Principe Felipe	Spain	16
4	University of Alberta	Canada	14
	Tel-Aviv University	Israel	14
	Max Planck Society for the Advancement of Science	Germany	14
5	Université Paris 7 Diderot	France	13
6	Boston University	USA	12
	Swiss Institute of Bioinformatics	Switzerland	12
7	Universität Göttingen	Germany	11
	University of Washington	USA	11
8	Universität Bielefeld	Germany	10
9	Centre National de la Recherche Scientifique	France	9
	National Institutes of Health	USA	9
	Academia Sinica	Taiwan	9
10	Agency for Science, Technology and Research	Singapore	8
	Boston College	USA	8
	Deutsches Krebsforschungszentrum	Germany	8
	European Bioinformatics Institute	UK	8
	Indian Institute of Science	India	8
	Institute of Microbial Technology	India	8
	National Taiwan University	Taiwan	8
	Stanford University	USA	8

Collecting the institution where each service is hosted allows us to count the number of times a specific institution occurs in our tables. This table sums up the top ten ranks for all institutions for the NAR *Web Server Issues* from 2003 to 2009.

#### Citations as a Measure of Success

During our assessment of each service, the final data point we recorded was the Web page's overall level of usability. Clearly arranged user interface widgets, default values, and easily accessible help and usage information give a service a high usability score. Crowded, unclear user interfaces without default values and harsh restrictions on admissible input data lead to a low usability score. To reduce subjectivity, services were rated by two individuals independently, and results averaged. Refer to Table 4 for more details.

**Table 4:** Properties and Features Considered for the Usability Score.

Qualities analyzed	points
Model service, intuitive user interface, presence of documentation,	
default values, examples, version and contact information	3
Average service, may be in violation of one of the points above	2
Service below average, more than one violation, cluttered interface,	
unable to start within a few clicks	1
Fatal flaw, almost all points violated	0

The scoring system for the usability score from 0 to 3, evaluated by two persons independently. For overlapping data, we calculated Pearson's cross-correlation coefficient at 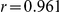.

**Figure 5:**
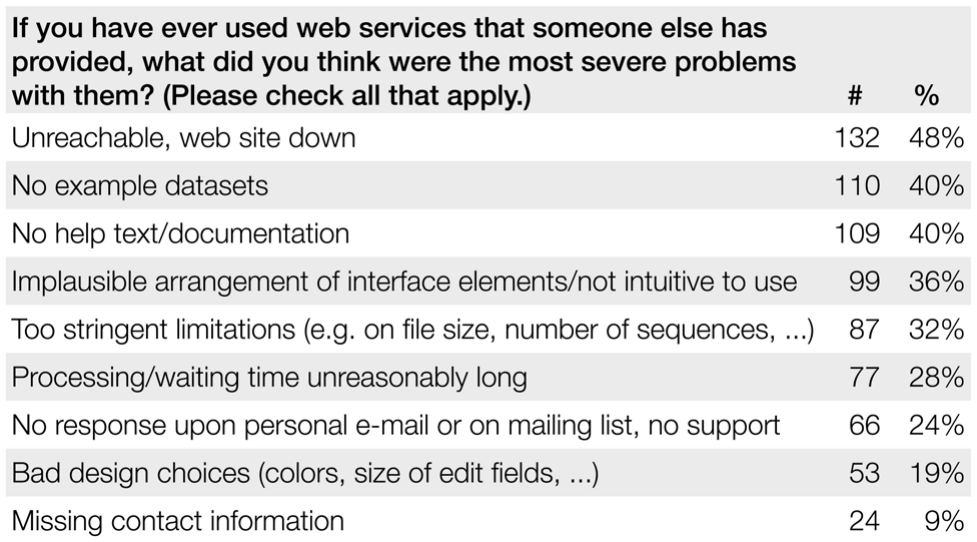
The main issues respondents have with other Web services. In our survey among 274 authors, we asked about problems using other bioinformatics Web services. The order of answers was randomized for each respondent and multiple answers were possible. Ranked in the first place is the users' main complaint, i.e., that the Web site hosting the service was not functional. We can therefore infer that users are willing to overlook other problems with a service as long as it is in fact functional.

To confirm the validity of such a seemingly subjective measure, we counted the number of citations that services with high usability attract and contrasted it with the number for services with low usability in Figure 1 L and M. The numbers show that a service with high usability receives on average 1.8 times the citations of a low-usability service (cf. Tables 4 and 5). See the section on the Author Survey for details on obtaining the number of citations for a service, and Figure 5 for the most severe problems users have with other researchers' Web services.

**Table 5:** Average Citations for Services with High and Low Usability from 2003 to 2009.

Year	Citations/High Usability	Citations/Low Usability
2003	46	18
2004	18	12
2005	13	10
2006	9	4
2007	6	9
2008	3	1
2009	1	0
Average	14	8

Contrasting the number of citations for services with a high score in usability (2–3) with available, but low-scoring services (see Table 4). On average, a service with high usability is cited 1.8 times more often than a service with low usability is.

Figure 3 C shows the average citations for NAR *Web Server Issue* articles for a given year. The total number of citations is not comparable in a fair way from year to year; older publications have had more opportunity to be circulated, noted, and cited. Intuitively, unusable services should have a lower number of citations. Unsurprisingly, there is a difference in number of citations between services whose Web sites we found available and those that were unavailable. This difference increases with the years since publication. For each set of columns in Figure 3 C, we show the percentage of services that were unavailable for the given issue as a red circle, for comparison.

**Figure 6:**
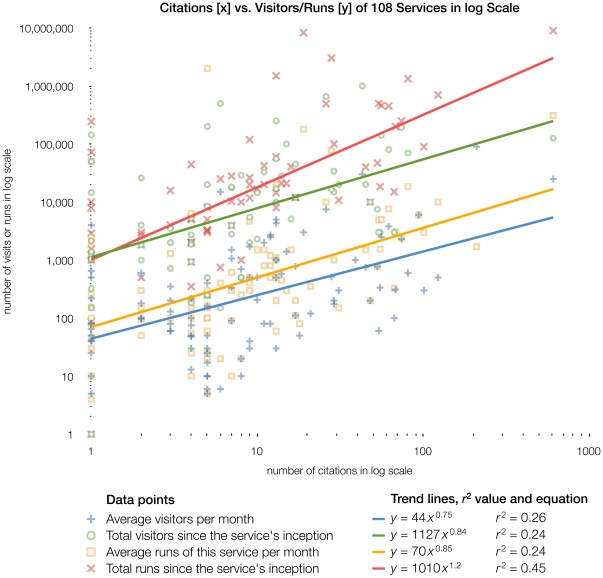
Number of citations listed in PubMed Central plotted against that service's number of visitors and runs. 108 of the 274 respondents (39%) to the author survey chose to answer the optional question about the Web address of their service and also gave numbers for monthly and total visits and runs. We combined this information with the number of times a service is cited in articles deposited in PubMed Central. Plotting these information against each other in log scale reveals relationships, shown as trend lines. The data points are based on the information given by the authors of the service themselves.

Data from the 2003 issue shows that even services that have been around for a long time are still used and cited. Tracking user statistics is thus more meaningful than mere age of a service in determining its usefulness. This is reflected in the extremely high number of visitors for articles from 2003 (see Figure 6).

### Author Survey

We conducted an anonymous survey consisting of six brief questions among the corresponding authors of 913 NAR *Web Server Issue* articles. Over 100 email addresses were no longer available. In that case, we tried to find contact information on the service Web site, but for unavailable services this rarely led to a valid address. Consequently, we suggest here that journals should allow authors to update their current email address for correspondence. In total, we sent 872 emails to authors asking for participation in the survey. We received 274 replies, a return rate of 31%.

#### Data on the Respondents' Own Services

Figure 2 illustrates the responses to the first question, about usage statistics of their service. 43% of respondents (119 in total) were not prepared to answer this question; we anticipated this because estimating usage statistics is inherently difficult and a problem any Web master is facing. Some countries (e.g. Germany) have laws against collecting information to uniquely identify Web site visitors, which makes many Web analytics tools illegal to use. The numbers reported by this subset of respondents should thus only be treated as an estimate. We asked about the respondents' reasons for not answering, offering several pre-defined, mutually exclusive choices. The number of respondents giving this answer is given in brackets: *Too much trouble to implement* 9% (26), *we don't collect statistic due to data privacy concerns* 6% (16), *we don't have access to this data, but it is collected by our institution* 4% (11), *don't know* 8% (21). Additionally, 24% of respondents added comments explaining why they could not answer the usage statistics question, mostly giving varying reasons why this information is not available to them. We charted visitor information against number of citations in Figure 6 to show that a heavily used service will also be cited more frequently.

**Figure 7:**
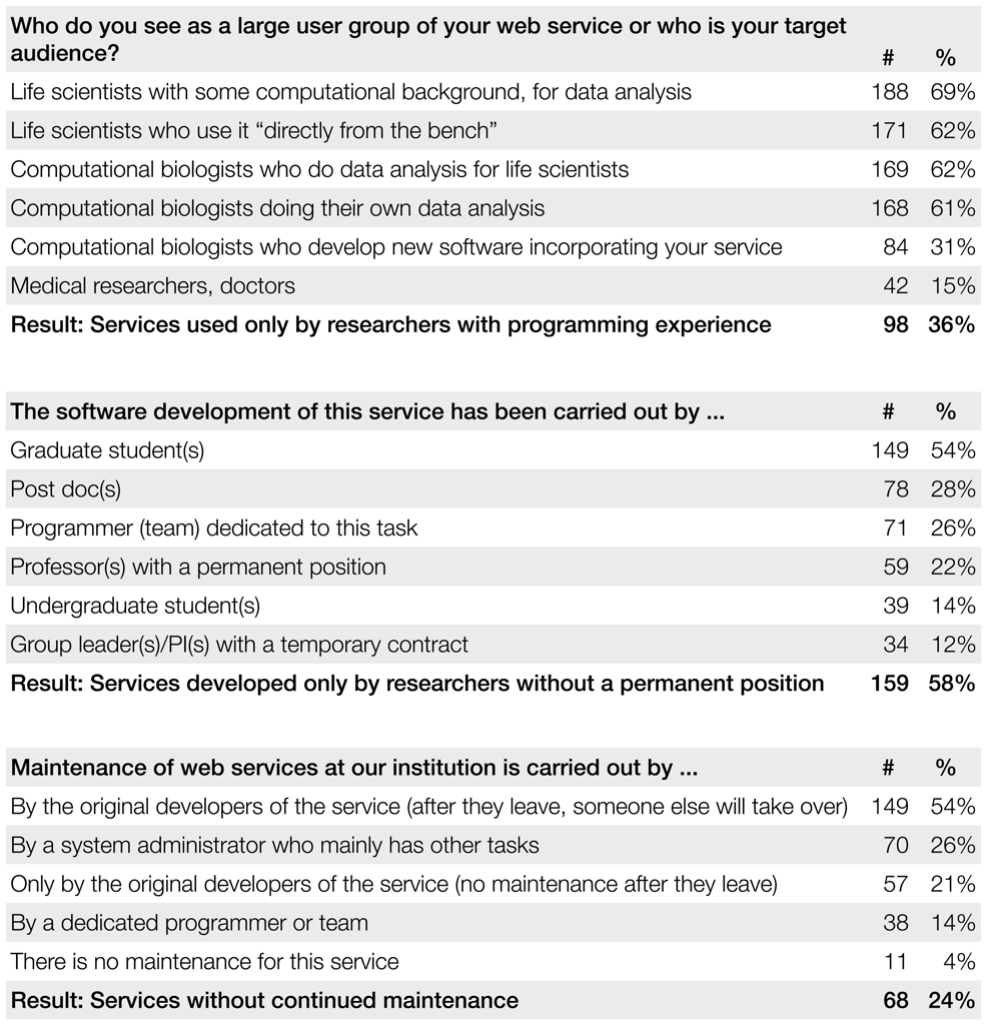
Target audience and the persons involved in development and maintenance of a Web service. In our survey among 274 authors, we asked about their service's expected users. Multiple answers were possible. The most interesting result of the first question is that Web services are indeed intended for researchers without programming experience, only 36% of services are used solely by researchers with a programming background, as estimated by the respondents. The second question indicates that services are rarely funded by separate grants, and development is carried out by researchers with a temporary position, such as graduate students, post-docs, etc., in 58% of all cases. The third question reveals that by the time of this survey, about 24% of services will soon lack persons maintaining them. 13 respondents (5%) indicated that they are going to maintain the service even after moving to another institution.

Three questions of our survey were about the services offered by the respondents themselves, as shown in Figure 7. For 64% of services, the projected target audience includes users without programming experience. Interface usability should be a primary concern when developing for this audience, because the service will be used from its Web interface; access of the service from within another program or a script commonly only requested and implemented by other computational biologists. Only 36% of respondents think their services are used exclusively by researchers with a background in programming. Clearly, most Web services are created for experimental scientists, while computational biologists often prefer stand-alone tools that can be integrated into workflows.

According to the respondents, most services (58%) has been developed without any help from researchers or programmers with a permanent position. For 54% of services, a successor has yet to be found to take over maintenance. It can be quite challenging to find someone to take over maintenance of an already published service. It is the senior author's responsiblity to figure this out early enough so there is enough transitional time and no extensive interruption in service.

For 24% of services, their authors already state that there will be no continued maintenance after the original developers leave for another position. The estimate of one quarter of services without maintenance closely matches the number of services from our study found to be no longer functional after more than three years of operation. We thus estimate that after three years from the last publication, 24% of services will be no longer maintained and eventually go offline.

#### Perceived Problems when Using External Services

Figure 5 shows the results for the question about perceived problems with other Web services. The users' main complaint (48%) was that the Web site hosting the service was not functional. This means that users are willing to overlook other problems with a service as long as it is in fact functional. A second set of problems indicated by the respondents pertains to some of the requirements that submissions to the NAR *Web Server Issues* now have to fulfill, namely example data sets (40%), usage help and documentation (40%), free access, and non-restrictive input size limits (32%). Missing contact information or lack of support was a problem for 33% of respondents, probably persons who have had a bad experience with unresponsive authors in the past [3].

We offered an open comment field for this question that some respondents used to describe specific, problematic scenarios: changes made to the service went undocumented and led to inconsistent results; lack of good default values; mandatory registration to access the service. In an extensive survey among users of bioinformatics databases for the ELIXIR project, Palcy and de Daruvar asked a similar question with very different answer options [14]. The question was entitled “Challenges with bioinformatics databases.” During their survey, users selected the option “database Web page usability” most often. The other options were quite database-specific. There were no answer options pertaining to functionality or availability of a Web page.

These perceptions are not too far from the observed problems in the Availability Study. While journals or funding agencies can enforce a set of rules, it still takes dedication to develop and maintain a Web service properly. Offering a Web-based application in addition to a research article is a very noble idea, but support for the software has to be offered as well.

The key figures of the Availability Study are shown in Table 1, including the number of responses we received for the author survey.

### Conclusion

We can learn from the availability study and the author survey above that disappearing Web services are a fact of life, no matter how stringently the submission instructions to authors are enforced. The scientific community has to develop some coping mechanisms to ensure the scientific record is preserved and future generations of scientists will be able to draw from the wealth of knowledge we have created.

#### Caveats of this Study

We elected to analyze a highly curated subset of all Web services ever published: only services described in any NAR *Web Server Issue*. This defined the scope of this study, but at first glance, it appears that the generalization is limited.

However, comparing our data set to the Bioinformatics Links Directory, which contains links to services “selected on the basis of recommendations from bioinformatics experts in the field,” [4], [5], we cover 74% of all services listed there (excluding databases), a very representative amount.

We consider the NAR *Web Server Issues* to be very well curated and edited. Nowhere else are peer reviewers so specifically instructed to enforce the strict requirements of the journal for publication of a manuscript dealing with a Web service. The authors and reviewers have a point-for-point checklist on qualities their services have to fulfill, and these are enforced stringently. Additionally, all submissions are pre-screened by the issue's editor. At the same time, the NAR Web Server Issue is quite lenient when it comes to something most other journals do not accept: re-publication of existing research. Previously published material may still be eligible for re-publication in the NAR *Web Server Issue*, because the editors want to be able to include all highly used and well-known services. Thus, the NAR *Web Server Issues* cover the best services in computational biology and any results we present here can be regarded as an upper bound in availability, quality, and maintenance that has been achieved in this field. Any other collection of Web services two to seven years old will most likely exhibit lower scores on availability, usability, and on the LT-Score scale.

Since even this data set is far from perfect, the question arises what can be done to remedy the status quo. There are three entities that can exert pressure on the main developers and authors of Web service publications. Below, we have compiled suggestions for supervisors, editors and reviewers, and finally for funding institutions.

#### Reproducibility and Repeatability

When a Web service is published, it becomes part of the scientific record. A researcher using the output from a Web service in another publication puts reproducibility into the hands of the service developer. Once the service becomes unavailable, the derived research becomes unverifiable. In most other scientific disciplines, something like that is unacceptable. See below for some suggestions what authors can do to avoid this problem.

#### Funding

Maintaining a Web service is still an undervalued, unpaid and rarely appreciated effort. Therefore, it has to become part of good scientific practice. Unfortunately, most funding agencies do not offer grants for the maintenance of already-established infrastructure. Using the results of this study, we hope to provide some weight for researchers seeking funds for their services. This could either be done by greatly expanding efforts such as the collection of services at government research institutions, or by increasing funding for projects like the BioCatalogue [15], Taverna [16], or Galaxy [17].

#### Responsibilities

It is the responsibility of the senior author to determine how Web service maintenance will be distributed. Either developers can take their work with them or it stays with the lab. Both require some planning ahead but clear-cut rules will go a long way.

#### Editors and Reviewers

As an editor or reviewer, one is in a unique position of power to impose rules upon submitters of manuscripts. This can be used for good when a sensible set of rules is enforced. As a reviewer, we suggest to visit any Web services mentioned in the manuscript and to try to submit some example data. One should make sure all the points expected from a Web service as a user are there, or use our LT-Score to evaluate the page. We have created an online tool for this task, available on our supplementary web page at http://bioweb.me/tl-score.

Many problems from unavailable Web services stem from the need to change its address eventually. If possible, provide authors with a DOI address for their service. Additionally, requiring the deposition of the application's source code in an open source software repository would make a great rule.

#### Suggestions

For developers and maintainers of Web services, we have compiled a list of ten simple rules that can be followed to make providing a scientific Web service much easier. See “Ten Simple Rules for Providing a Scientific Web Service” [13] and the summary below.

- Start out by choosing a good name and getting a permanent URL for that, for example register an internet domain name for the service or use our link referal service at http://bioweb.me
- clarify responsibilities with the project's supervisor, think about whether it is possible to take this work along or leave it with the lab
- consult the potential users of the Web service and let them know what can and cannot be achieved in a reasonable time frame
- check with collaborators, local system administrators, etc. to find a good way to host the service – it is great to use already available resources for that
- if it is not yet decided which programming language and framework to use, take a look at some of the features that e.g. Galaxy or Taverna have to offer
- make sure the software can run on more than one computer: it will have to be moved somewhere else sooner or later
- ideally, create an open source project at a place like [18] or [19] for the service, where all collaborators, users and future developers can work together on the project
- provide users with enough documentation and example data to get them started, and continue to support them when they have questions
- create a mailing list, blog, bug tracker and/or FAQ page with announcements; this comes free when starting a project at an open source software site
- in the output of the service, give users everything they need to run the experiment again if need be, thus facilitating reproducibility of their research
- plan ahead to hand over maintenance to somewhere else, that means documented code and some installation or build instructions
- if it becomes clear there are no more active users or the service cannot be maintained any longer, it is time to switch it off – release the final version of the source code once more or create a virtual machine from the server running it

By following these suggestions, authors will increase the chance that their service is available and usable for all who will find it useful. If our suggestions become part of good scientific practice, it is our conviction that source code and service quality will increase and the whole community of computational biologists will benefit.

## Methods

### Web Service Availability

We visited all Web services that are listed in the NAR *Web Server Issues* from 2003 to 2009 [1], [20]–[25] from June to October 2009, and again in August and October 2010. We recorded whether the address is redirected or changed. If a page was unavailable, we searched for the service name and if that was ambiguous, also for the authors' names, in internet search engines, to locate a newer page, if one existed. If there were no results or those, too, were unavailable, the service is marked as unavailable in our Dataset S1. Please refer to Dataset S2 for the Web addresses originally extracted from the abstracts.

The evaluation criteria were the presence or availability of: a working, available URL; a visible version number and indication of the last update; contact information; access without registration; Web-based form; example data; help and usage information; a “fair” opportunity for us to test the service; the number of citations in PubMed Central. These criteria are collected from journals' requirements, software best practices, and our own experience. Services were evaluated by three individuals, and for overlapping data we calculated Pearson's cross-correlation coefficient at 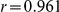.

A Web service is deemed available if accessing its URL did not return an error message (as described in [3]). To determine if our snapshot visits only reflect a temporary downtime of some of the services, we tried to access unavailable services again about one month after the initial visit and again one year after that. Available services may show their regular Web page but could still be nonfunctional. To investigate this, we checked for a “fair” testing possibility.

A “fair” testing possibility is given if the service has a one-click test functionality, where example data is entered e.g. via JavaScript. Furthermore, we also considered it fair if the service provided example input files for download or only required common file formats such as FASTA/Q, GFF/3, PDB, BED, CSV, TXT, and XML. For a few servers, incomplete input specification or lack of example data prevented us from testing them, thus we could not confirm their operational status.

These criteria are combined in a measure we call the LT-Score for Web service quality assessment. This is a sum of all scores we assign to each of these criteria. Separate scores exist for Web services and collections of services. Refer to Table 2 for details on calculating the LT-Score. Details about the usability scoring can be found in Table 4. Complete study and survey data can be found online in the Dataset S3.

The citation count was taken from PubMed Central in January 2010 and serves only as an indicator of the total number of citations, as not all relevant journals are deposited in PubMed. The country of the Web server was determined using the Mozilla Firefox browser add-on Flagfox [26], using mapping data of a server's IP address to a physical location (geolocation) provided by MaxMind, Inc. The countries and institution hosting the service were recorded. For the top-ranking countries and institutions, see Table 3 and Figure 4.

### Survey Among Authors

We tried to contact each corresponding author of an NAR *Web Server Issue* publication for a short survey. We asked them to provide information about: number of users per month; if no usage statistics are collected, why not; common problems when using other bioinformatics Web services; development, maintenance and planned handing-over of the service published in NAR; expected target audience. Using specific links to the survey, we were able to separate all answers according to year of publication.

The questions were multiple choice, the order of choices was randomized individually. Multiple answers were allowed. We anonymized the answers by discarding any personal data generated by accessing the survey Web page. Some respondents volunteered the Web address of their service, in which case we mapped the number of visitors to the number of citations to create Figure 6.

In some cases, the corresponding author was not reachable under the listed email address. We then tried to use the contact information given on the service's Web site. Despite our efforts, not all authors could be contacted.

A complete list of questions and the answers provided can be found in the Dataset S3.

## Supporting Information

Dataset S1 **Main Web services data points tables.** Tables, by year, of all data collected on the services studied in the manuscript. All fields used to calculate the LT-Score for each of the services are included. For the original Web addresses extracted from the abstracts, refer to Dataset S2.
(XLS)

Dataset S2 **Original Web addresses from abstracts.** This list contains the original Web addresses extracted from the abstracts of the NAR *Web Server Issues*.
(XLS)

Dataset S3 **Complete data tables for Web services and author survey.** Complete evaluation data and replies from the survey among authors on several spreadsheets.
(XLS)

## References

[1] Roberts RJ (2003). Editorial.. Nucl Acids Res.

[2] Benson G (2010). Submitting to the web server issue.. http://www.oxfordjournals.org/our_journals/nar/for_authors/submission_webserver.html.

[3] Veretnik S, Fink JL, Bourne PE (2008). Computational biology resources lack persistence and usability.. PLoS Comput Biol.

[4] Brazas MD, Yim DS, Yamada JT, Ouellette BF (2011). The 2011 bioinformatics links directory update: more resources, tools and databases and features to empower the bioinformatics community.. Nucleic Acids Res.

[5] Brazas MD, Yim DS, Yamada JT, Ouellette BF (2011). Bioinformatics Links Directory.. http://bioinformatics.ca/links_directory.

[6] Sietmann R (2009). Open science: Rip. Mix. Publish.. Magazin für Computer Technik.

[7] Thornton J (2009). Data curation in biology – past, present and future.. Nature Precedings.

[8] Thornton J, BioSapiens Network (2009). Annotations for all by all - the biosapiens network.. Genome Biol.

[9] McWilliam H, Valentin F, Goujon M, Li W, Narayanasamy M (2009). Web services at the european bioinformatics institute-2009.. Nucleic Acids Res.

[10] McGinnis S, Madden TL (2004). Blast: at the core of a powerful and diverse set of sequence analysis tools.. Nucleic Acids Res.

[11] Sayers EW, Barrett T, Benson DA, Bolton E, Bryant SH (2010). Database resources of the national center for biotechnology information.. Nucleic Acids Res.

[12] Tao T (2010). Analysis tool portfolio – how to get in? Personal Communication, NCBI Help Desk Email HD-3446.. Computational Biology Web Service Availability.

[13] Schultheiss SJ (2011). Ten simple rules for providing a scientific web resource.. PLoS Comput Biol.

[14] Palcy S, de Daruvar A (2009). Elixir bioinformatics user survey..

[15] Bhagat J, Tanoh F, Nzuobontane E, Laurent T, Orlowski J (2010). BioCatalogue: a universal catalogue of web services for the life sciences.. Nucl Acids Res.

[16] Hull D, Wolstencroft K, Stevens R, Goble C, Pocock MR (2006). Taverna: a tool for building and running workows of services.. Nucleic Acids Res.

[17] Goecks J, Nekrutenko A, Taylor J, The Galaxy Team (2010). Galaxy: a comprehensive approach for supporting accessible, reproducible, and transparent computational research in the life sciences.. Genome Biol.

[18] Geeknet, Inc (2010). Sourceforge.net: Open source software development web site.. http://sourceforge.net/.

[19] Bioinformatics Organization (2010). Collaborative development environment.. http://www.bioinformatics.org/wiki/Hosting.

[20] Roberts RJ (2004). Editorial.. Nucl Acids Res.

[21] Roberts RJ (2005). Editorial.. Nucl Acids Res.

[22] Roberts RJ (2006). Editorial.. Nucl Acids Res.

[23] Benson G (2007). Editorial.. Nucl Acids Res.

[24] Benson G (2008). Editorial.. Nucl Acids Res.

[25] Benson G (2009). Editorial: Nucleic acids research annual web server issue in 2009.. Nucl Acids Res.

[26] Garrett D (2010). Flagfox.. http://flagfox.net/.

[27] Tufte ER (2006). Beautiful Evidence.

